# Recruitment of *Saccharomyces cerevisiae* Cmr1/Ydl156w to Coding Regions Promotes Transcription Genome Wide

**DOI:** 10.1371/journal.pone.0148897

**Published:** 2016-02-05

**Authors:** Jeffery W. Jones, Priyanka Singh, Chhabi K. Govind

**Affiliations:** Department of Biological Sciences, Oakland University, Rochester, MI-48309, United States of America; Southern Illinois University School of Medicine, UNITED STATES

## Abstract

Cmr1 (changed mutation rate 1) is a largely uncharacterized nuclear protein that has recently emerged in several global genetic interaction and protein localization studies. It clusters with proteins involved in DNA damage and replication stress response, suggesting a role in maintaining genome integrity. Under conditions of proteasome inhibition or replication stress, this protein localizes to distinct sub-nuclear foci termed as intranuclear quality control (INQ) compartments, which sequester proteins for their subsequent degradation. Interestingly, it also interacts with histones, chromatin remodelers and modifiers, as well as with proteins involved in transcription including subunits of RNA Pol I and Pol III, but not with those of Pol II. It is not known whether Cmr1 plays a role in regulating transcription of Pol II target genes. Here, we show that Cmr1 is recruited to the coding regions of transcribed genes of *S*. *cerevisiae*. Cmr1 occupancy correlates with the Pol II occupancy genome-wide, indicating that it is recruited to coding sequences in a transcription-dependent manner. Cmr1-enriched genes include Gcn4 targets and ribosomal protein genes. Furthermore, our results show that Cmr1 recruitment to coding sequences is stimulated by Pol II CTD kinase, Kin28, and the histone deacetylases, Rpd3 and Hos2. Finally, our genome-wide analyses implicate Cmr1 in regulating Pol II occupancy at transcribed coding sequences. However, it is dispensable for maintaining co-transcriptional histone occupancy and histone modification (acetylation and methylation). Collectively, our results show that Cmr1 facilitates transcription by directly engaging with transcribed coding regions.

## Introduction

The *S*. *cerevisiae* protein Ydl156w/Cmr1 (changed mutation rate 1) is a putative WD40 domain containing protein [1], with suggested homology to two human proteins: Ddb2 (DNA damage binding protein 2) and Wdr76 (WD repeat protein 76), based on sequence similarity [2]. Several studies have suggested a role for Cmr1 involvement in DNA damage/repair and replication stress response.

Initially identified in a screen for altered sensitivity to Tirapazamine (an anticancer drug) [3], the *cmr1*Δ mutation was among those showing a marked increase in resistance to Tirapazamine, which under hypoxic conditions is converted into radical species inducing DNA damage leading to cell death [4]. It was also found in a genome-wide screen that in response to methyl methanesulphonate (MMS) and hydroxyl urea (HU) treatment (induces DNA damage and replication stress) Cmr1 localizes to sub-nuclear foci. These foci contained many proteins involved in DNA repair, suggesting that Cmr1 might play a role in preventing DNA damage or in overcoming replication stress [5]. Interestingly, the Cmr1 focus was distinct from the canonical Rad52 DNA repair focus [5], suggesting that it may perform a different function than Rad52 or its associated proteins in regulating genome integrity.

More recently, it was shown that in response to replication stress Cmr1, along with 27 proteins, forms an intranuclear quality control compartment, which contains misfolded, ubiquitylated and sumolyated proteins [6]. Interestingly, proteins co-localizing with Cmr1 included the histone deacetylases Hos2 and Rpd3, which are implicated in deacetylating nucleosomes in transcribed coding sequences [5, 6]. Furthermore, *in vitro* studies have shown that recombinant Cmr1 binds preferentially to UV-damaged DNA and co-purifies with the chromatin fraction of UV-irradiated cells [2]. These studies further support a role for Cmr1 in DNA-damage response. Accordingly, *in silico* analyses revealed that Cmr1 expression clusters with proteins involved in the DNA repair pathway [7]. These studies, therefore, suggest a role for Cmr1 related to DNA damage/repair and replication stress.

A recent study utilizing tandem affinity purification (TAP) coupled with mass spectrometry and multidimensional protein identification technology (Mud-PIT) identified Cmr1 as one of the core components of histone interacting proteins [8]. Reciprocal Mud-PIT analysis of Cmr1 confirmed its interaction with all four histones, and also with many proteins involved in DNA recombination, repair and replication. Interestingly, it also showed interactions with chromatin remodelers (SWI/SNF and RSC), histone modifying complexes such as the histone acetyltransferase SAGA and the histone deacetylase Rpd3, as well as with the FACT complex subunits (Spt16/pob3). While many of these factors play a role in DNA damage response, they are also critically important for Pol II-mediated transcription [9, 10]. Remarkably, while Mud-PIT analyses revealed Cmr1 interaction with many subunits specific to Pol I and Pol III RNA polymerases (reviewed in [11, 12]), no interaction was observed with Pol II, which transcribes all protein-coding genes [8, 13]. However, interaction of Cmr1 with the proteins involved in Pol II-mediated transcription, such as the Paf1 complex [14, 15] was observed. Chromatin remodelers and histone modifying complexes identified as Cmr1-interacting partners are intricately involved in regulating Pol II transcription [16–19]. These observations suggest that Cmr1, in addition to regulating chromatin during replication or DNA-damage stress, may also play a role in Pol II mediated transcription.

In this study, we have examined the recruitment and function of Cmr1 at Pol II transcribed genes. We show, for the first time, that Cmr1 is recruited to transcribed coding regions, but not to the promoters, of Gcn4 and Gal4 regulated genes in a transcription-dependent manner. ChIP-chip analysis revealed that Cmr1 is recruited to many coding sequences, genome-wide. Furthermore, we provide evidence that Cmr1 recruitment to coding regions is stimulated by the Pol II CTD kinase Kin28 as well as by the histone deacetylases Rpd3 and Hos2. Pol II occupancy in a *cmr1*Δ mutant was reduced at the coding regions of many highly transcribed genes, including Gcn4 targets and ribosomal protein genes. Thus our study suggests that cotranscriptional recruitment of Cmr1 to coding regions promotes transcription at the elongation step.

## Materials and Methods

### Growth conditions

*S*. *cerevisiae* strains were cultured in synthetic complete media lacking amino acids isoleucine and valine (SC^-ILV^). For inducing Gcn4 target genes (*ARG1* and *HIS4*), the cultures were grown to an optical density of 0.5–0.6 measured at 600 nm (A_600_), and treated with 0.6 μM of sulfometuron methyl (SM) for 30 minutes. To inactivate Ser5 kinase, *kin28as* cells were treated with NA-PP1 (6 μM) for 15 minutes. *bur1as* cells were treated by 3MB-PP1 (6 μM) for 30 minutes to inactivate Bur1 prior to induction of Gcn4 targets by SM. For inducing *GAL1*, 2% galactose was added to the cells grown to an A_600_ of 0.5–0.6 in yeast extract-peptone-raffinose (YPR). To repress *GAL1* transcription, 4% glucose solution was added to the galactose-induced cells for 10 minutes.

### Yeast strains

All *S*. *cerevisiae* strains used in this study were procured from ThermoScientific, and the list of strains is provided in the S1 Table. Myc-tagged strains were generated by a PCR-based method as described previously [20]. Briefly, plasmid pFA6a-13Myc-HIS3MX6 was used as a template to PCR-amplify the 13Myc-*HIS3* cassette using primers encompassing sequences homologous to regions upstream and downstream of the stop codon of the gene of interest. The amplified DNA was used for transforming various deletion strains, and the colonies positive for integration were confirmed by PCR, and the expression of Myc-tagged protein was confirmed by western blot analysis.

### Chromatin immunoprecipitation

Chromatin immunoprecipitation assays were performed as described previously [21]. Briefly, 100 ml of induced cultures were cross-linked with 1% formaldehyde solution for 15 minutes at room temperature, and the cross-linking reaction was quenched by adding 15 ml of 2.5 M glycine. Chromatin was sheared to an average size of 250–350 base pairs fragments by sonication (Branson 450), and the soluble chromatin was subjected to immunoprecipitation using antibodies against Myc (Roche, 116672030010), Rpb3 (Neoclone, W0012), histone H3 (Abcam, ab1791), H3Ac (Millipore, 06–599), H3K36me2 (Abcam, ab9049), H3K36me3 (abcam, ab9050), H3K4me2 (Millipore, 05–1338), H3K4me3 (Millipore, 05–745), phospho-Ser5 (Covance, MMS-134R), and phosphor-Ser2 (Bethyl laboratories, A300-654A). Input and ChIP DNA was amplified using primers specific to the promoter and coding regions of *ARG1*, *HIS4*, *GAL1*, *PMA1* and *ADH1* genes. Primers against the *POL1* ORF were included in the PCR reactions as an internal control for immunoprecipitation. PCR-amplified DNA was stained with SYBR-green dye (Lonzo Inc., 50513), resolved on 8% TBE gels, and signals were quantified by phosphorimager using ImageQuant software (Molecular Dyanamics). The relative fold enrichment was determined by taking a ratio of the signals obtained for the specific gene regions and *POL1* for ChIP DNA, and normalized against the input DNA/*POL1* signal ratio. For measuring histone modifications, a region of *TELVI* was used as the internal control. The primers used are provided in S2 Table. All ChIP experiments were performed with at least three culture replicates and PCR reactions we conducted in duplicate. Errors bar represents standard error of mean (SEM).

### ChIP-chip experiments and analysis

The ChIP-chip experiments were performed as described previously [19]. Briefly, Cmr1 and Rpb3 ChIP DNA and corresponding input DNA from WT, *cmr1*Δ or *gcn4*Δ were amplified using the GenomePlex complete whole-genome amplification kit (Sigma, WGA2) as per manufacturer instructions. The ChIP and input DNA were labeled by Alexa555 and Alexa647, respectively using BioPrime Plus Array GCH Labelling System (Invitrogen, 18095–013). Equal quantity of labeled ChIP and input DNA were hybridized and processed as per the manufacturer instructions. The arrays were scanned on G2505C Agilent SureScan microarray scanner and data was extracted using Agilent Feature Extraction software.

The feature-extracted data was read into R software and normalized using Limma package from Bioconductor as described previously [19]. The ORF occupancies of Cmr1 and Rpb3 were determined by averaging normalized ChIP/input log_2_ values for the probes present within the transcription start site (TSS) and the transcription end sites (TES). Gene-average profiles were generated using the versatile aggregate profiler [22] as described previously [23].

### TCA precipitation and western blot analyses

Whole cell extracts for analyzing histone modification were prepared by the TCA (Trichloroacetic acid) extraction method as described previously [23]. Extracts were resolved on 10% SDS-polyacrylamide gels and transferred to nitrocellulose membrane. Proteins were detected by western blot using antibodies against H3, H3Ac, H3K14Ac, H3K18Ac, H3K23Ac, H4Ac or Gcd6 (a translation protein).

## Results

### Cmr1 is recruited to the transcribed coding sequences of Gcn4 and Gal4 target genes

A majority of studies suggest a role for Cmr1 in DNA damage and the replication-stress pathway. Recently, however, Cmr1 has been shown to interact with histones and various transcription factors, including chromatin remodelers (RSC and SWI/SNF), the histone deacetylase Rpd3, and the Paf1 complex [8]. Surprisingly, however, no interaction was observed with Pol II-specific subunits, while interactions with subunits specific to Pol I and Pol III were observed. Microarray analysis in *cmr1*Δ revealed altered expression of ~400 genes, including those that are transcribed by Pol I and Pol III [8].

**Fig 1:**
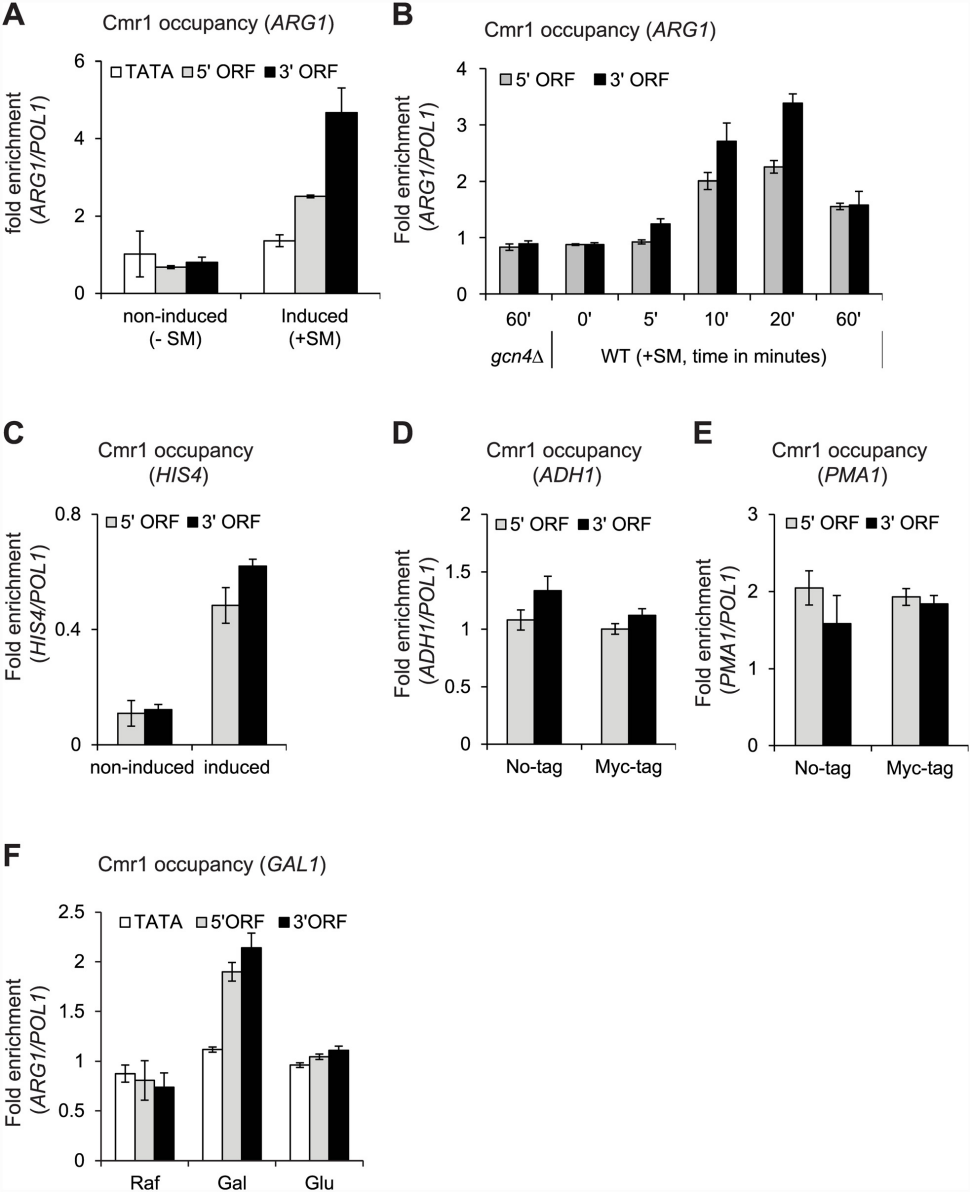
Cmr1 is recruited to the transcribed coding sequences of Gcn4 and Gal4 target genes. Cmr1-Myc tagged strains were treated with SM (0.6 μM) to induce Gcn4-target genes and processed for chromatin immunoprecipitation (ChIP) using anti-Myc antibodies. A) Cmr1 ChIP occupancies at the *ARG1* TATA, 5’ and 3’ ORFs are shown under Gcn4 non-inducing (-SM) and inducing conditions (+ SM). B) Cmr1 occupancy was measured at various time-points (0 min to 60 min) during the course of *ARG1* induction (+SM). Cmr1occupancy in a *gcn4*Δ at 60 minutes, post-induction is also shown. C) Cmr1 occupancy at the *HIS4* 5’ and 3’ ORFs under non-inducing and inducing conditions. D-E) Cmr1 occupancies at the 5’ and 3’ ORFs of *ADH1* (D) and of *PMA1* (E) are shown. Since both *ADH1* and *PMA1* are constitutively expressed, untagged WT strain was used as a control. F) Cells were grown in raffinose (Raf) and *GAL1* transcription was induced by adding 2% galactose (Gal) and repressed by adding 4% glucose (Glu). Cmr1 occupancy was determined at the *GAL1* TATA, 5’ and 3’ ORFs.

To address the possibility of a role in Pol II mediated transcription, we first asked whether Cmr1 associates with Pol II transcribed genes. Towards this end, we examined Cmr1 occupancy at the amino acid biosynthetic genes, which are strongly induced under amino acid starvation stress conditions [24, 25]. Yeast cells were treated with sulfometuron methyl (SM), an inhibitor of isoleucine/valine (ILV) biosynthesis [25, 26], to mimic amino acid starvation conditions, and the occupancy of Myc-tagged Cmr1 (referred to as Cmr1 hereafter) was measured at *ARG1* by chromatin immunoprecipitation (ChIP) assay. Under non-inducing conditions (-SM), there was no significant difference in Cmr1 occupancies observed at the *ARG1* promoter (TATA), or the 5’ and 3’ open reading frames (ORFs) (Fig 1A). In contrast, upon *ARG1* induction (+SM), Cmr1 occupancy increased significantly at the 5’ and 3’ ORFs, peaking in the 3’ ORF, indicating that Cmr1 is actively recruited to the coding regions of *ARG1* during transcription.

To further address transcription-dependent recruitment, we examined Cmr1 occupancy at various time points during the course of *ARG1* induction. We observed that Cmr1 occupancy increased with time and peaked between 10–20 minutes post-induction (Fig 1B). Cmr1 occupancy in *gcn4*Δ cells induced for 60 minutes was similar to that observed in the 0′ minute time-point (Fig 1B), consistent with the fact that *ARG1* transcription requires Gcn4 [26]. Similar to *ARG1*, increased occupancy of Cmr1 in the coding regions (5’ and 3’ ORFs) of *HIS4* was observed only under the inducing conditions (Fig 1C). To test whether Cmr1 is generally recruited to coding regions, we measured Cmr1 occupancy at two constitutively expressed genes, *ADH1* and *PMA1*. The occupancy of Cmr1 at these genes was similar to that seen in the untagged strain (Fig 1D and 1E), suggesting that Cmr1 is not recruited to house-keeping genes. Our data suggest that Cmr1 is differentially recruited to the ORFs of inducible genes (*ARG1* and *HIS4*) upon activation.

To further test this idea, we analyzed Cmr1 occupancy at *GAL1* (a Gal4 regulated gene), which is induced when cells are grown in the presence of galactose as a carbon source. As expected, Rpb3 occupancy (Pol II subunit) was substantially increased at the *GAL1* promoter (TATA) as well as at the 5’ and 3’ ORFs upon galactose (Gal) induction relative to that observed under the non-inducing conditions (raffinose, Raf) (S1 Fig) [17, 27]. Furthermore, Pol II occupancies at the promoter and ORF regions were greatly reduced upon addition of glucose (Glu), which strongly represses *GAL1* transcription (S1 Fig). In contrast to a significant increase in Pol II occupancy at the *GAL1* TATA upon induction, Cmr1 occupancy at this region was only slightly more than that observed under non-inducing condition (Fig 1F). However, a significant increase in Cmr1 occupancy was observed at the 5’ and 3’ ORFs of *GAL1* upon induction, indicating that Cmr1 is recruited only to the coding regions of transcribing *GAL1* gene. Importantly, Cmr1 occupancy was reduced to the non-induced levels within 4 minutes of glucose addition to the Gal-induced cells (Fig 1F and S1 Fig). Therefore, our results provide evidence for a direct association of Cmr1 with the Pol II transcribed genes in a manner dependent on transcription.

### Transcription-dependent recruitment of Cmr1 to coding regions

**Fig 2:**
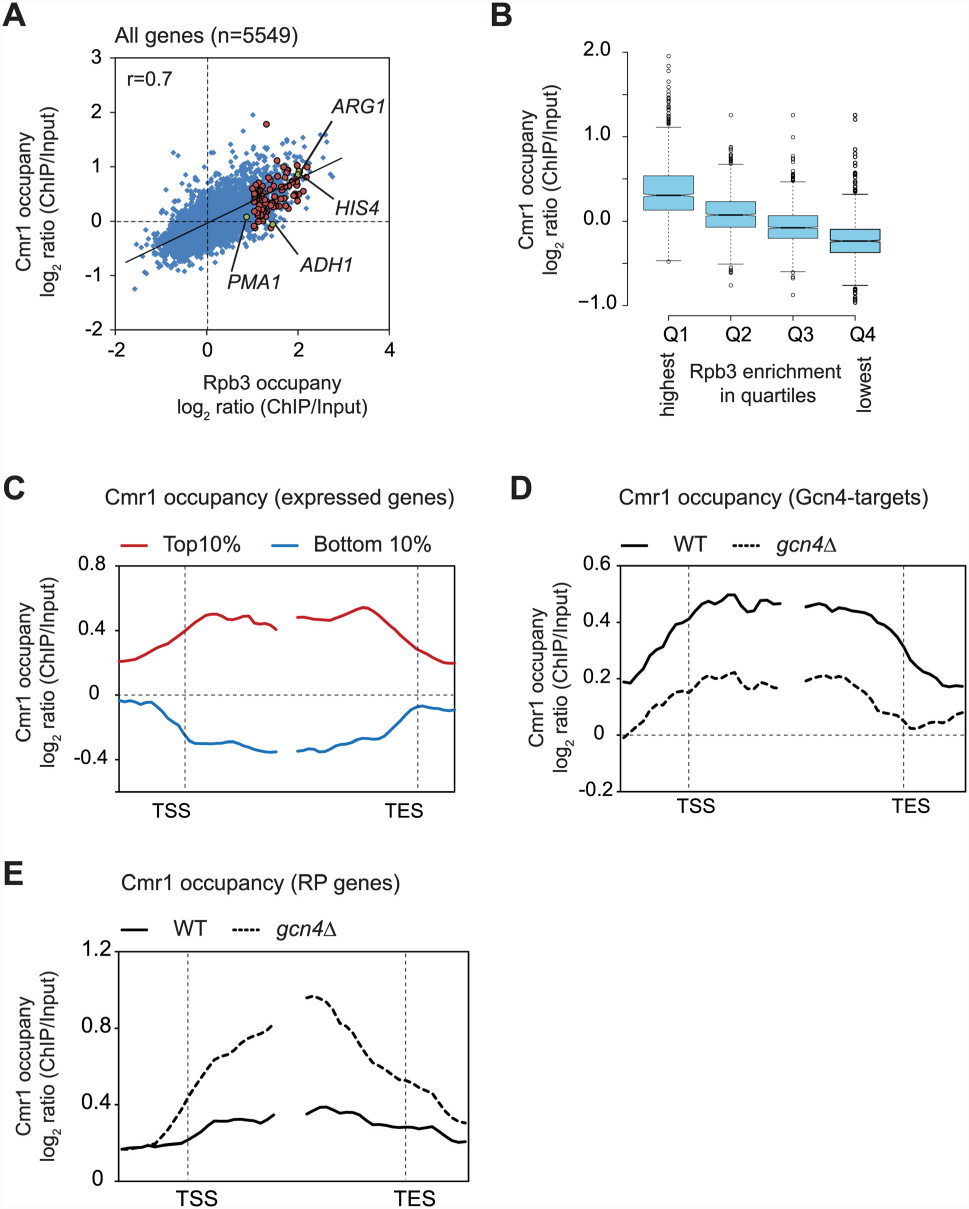
Transcription-dependent recruitment of Cmr1 to coding regions. A) Scatter plot showing correlation between Rpb3 and Cmr1 occupancies (averaged over ORF) in WT cells. The data points representing Gcn4 targets are shown by red circles, and *ARG1*, *HIS4*, *ADH1* and *PMA1* are marked (green circles) in the plot. B) Quartiles were generated based on average Rpb3 ORF occupancies (n = 1387, each quartile) and Cmr1 occupancies in each quartile are presented as a box-plot. Genes in quartile Q1 showed the greatest Rpb3 occupancy, and Q4 showed the least. Center lines show the medians; box limits indicate the 25th and 75th percentiles as determined by R software, and outliers are represented by dots. The notches represent 95% confidence intervals for each median. C) Cmr1 occupancy is shown at the metagene comprised of the top 10% and the bottom 10% of genes showing the greatest and the lowest Rpb3 occupancies, respectively. D) Average Cmr1 occupancy profile for the 94 Gcn4 targets harboring Rpb3 occupancy >1.0 log_2_ ratio (ChIP/input) is shown in WT and *gcn4*Δ cells. E) Cmr1 average occupancy profile at ribosomal protein (RP) genes (n = 135) is shown for the WT and *gcn4*Δ cells.

To better understand the genome-wide distribution of Cmr1 occupancy and its relationship with transcription, we performed Cmr1 and Pol II (Rpb3) ChIP-chip analysis using Agilent 4x44K arrays under amino acid starvation condition (+SM). For each gene, the average Cmr1 and Pol II occupancies were determined by averaging the signal intensities (normalized log_2_ ratio ChIP/input) of the probes present within the transcription start site (TSS), and the transcription end site (TES) of a given gene. Consistent with our results above (Fig 1), Cmr1 enrichments were high in the ORFs of *ARG1* (0.91 log_2_ ratio) and *HIS4* (0.85 log_2_ ratio), and low in the ORFs of *PMA1* (0.07 log_2_ ratio) and *ADH1* (-0.05 log_2_ ratio) (Fig 2A). Moreover, Cmr1 occupancy correlated with Pol II occupancy (Pearson correlation r = 0.7) in coding regions genome-wide, suggesting that the presence of Cmr1 in the coding regions requires transcription. To provide further support for this observation, we generated Rpb3 occupancy quartiles and asked which quartile shows greatest Cmr1 enrichment. This analysis revealed that the highest enrichment for Cmr1 was observed in the quartile (Q1) of genes exhibiting the greatest Rpb3 occupancies (Fig 2B). Metagene, comprised of the top 10% Pol II-occupied genes (n = 555), displayed uniform Cmr1 enrichment across the coding region (Fig 2C). In contrast, the coding region of the metagene comprised of the bottom 10% genes was depleted of Cmr1 (Fig 2C). These results reinforce the idea that Cmr1 is primarily recruited to the coding sequences of transcribed genes.

To further support this idea, we examined Cmr1 occupancy in a *gcn4*Δ strain treated with SM, which induces transcription of Gcn4 target genes. In WT cells, 94 Gcn4-targets genes exhibited average Pol II occupancies greater than the log_2_ ratio of 1.0 (strongly transcribed). Metagene comprised of these 94 genes revealed lower Cmr1 occupancy in *gcn4*Δ than that seen in the WT cells (Fig 2D). This result indicates that Cmr1 is recruited to ORFs of Gcn4-targets upon transcriptional activation. While SM-treatment activates Gcn4-regulated genes, it represses expression of ribosomal protein (RP) genes [28]. Under these conditions, Gcn4 binds to Rap1 (repressor and activator protein), and represses expression of RP genes [28–30]. If Cmr1 occupancy is truly responsive to transcriptional changes, an increased association of Cmr1 to the ORFs of RP genes is expected in a *gcn4*Δ strain. As expected, a greater Cmr1 occupancy was observed at the ORFs of these genes in *gcn4*Δ cells compared to WT cells (Fig 2E). Collectively, our genome-wide results strongly suggest that Cmr1 is actively recruited to coding sequences of many genes in a manner dependent on transcription.

### Ser5 kinase Kin28 but not Ser2 kinases recruits Cmr1 to the *ARG1* ORF

Having determined that Cmr1 localizes to the coding regions of transcribed genes, we next investigated the mechanism by which it is recruited. We first focused on evaluating the role of elongating Pol II on Cmr1 recruitment. The C-terminal domain (CTD) of Rpb1, the largest subunit of Pol II, is phosphorylated at Ser5 residue by the TFIIH associated kinase Kin28 [31, 32]. This phosphorylation marks the transition of Pol II from initiation to the elongation step [33, 34]. Phosphorylated Pol II CTD acts as a scaffold to recruit several elongation, mRNA processing and termination factors [17, 18, 32, 35–38]. Since we observed Cmr1 enrichment in coding regions, we asked if its recruitment is stimulated by phosphorylation of Ser5 (Ser5P). To this end, we determined Cmr1 occupancy in a *kin28as* (as; analog sensitive) strain, in which the Kin28 activity can be rapidly inhibited by treating cells with an ATP analog NA-PP1 [17, 18]. In addition to Ser5P, Ser2 is also phosphorylated (Ser2P) at the promoter, albeit to a lower level by Bur1/Bur2 [37]. To understand whether Ser5 and Ser2 phosphorylation cooperates in recruiting Cmr1, we additionally determined its occupancy in a *kin28as*/*bur2*Δ double mutant.

**Fig 3:**
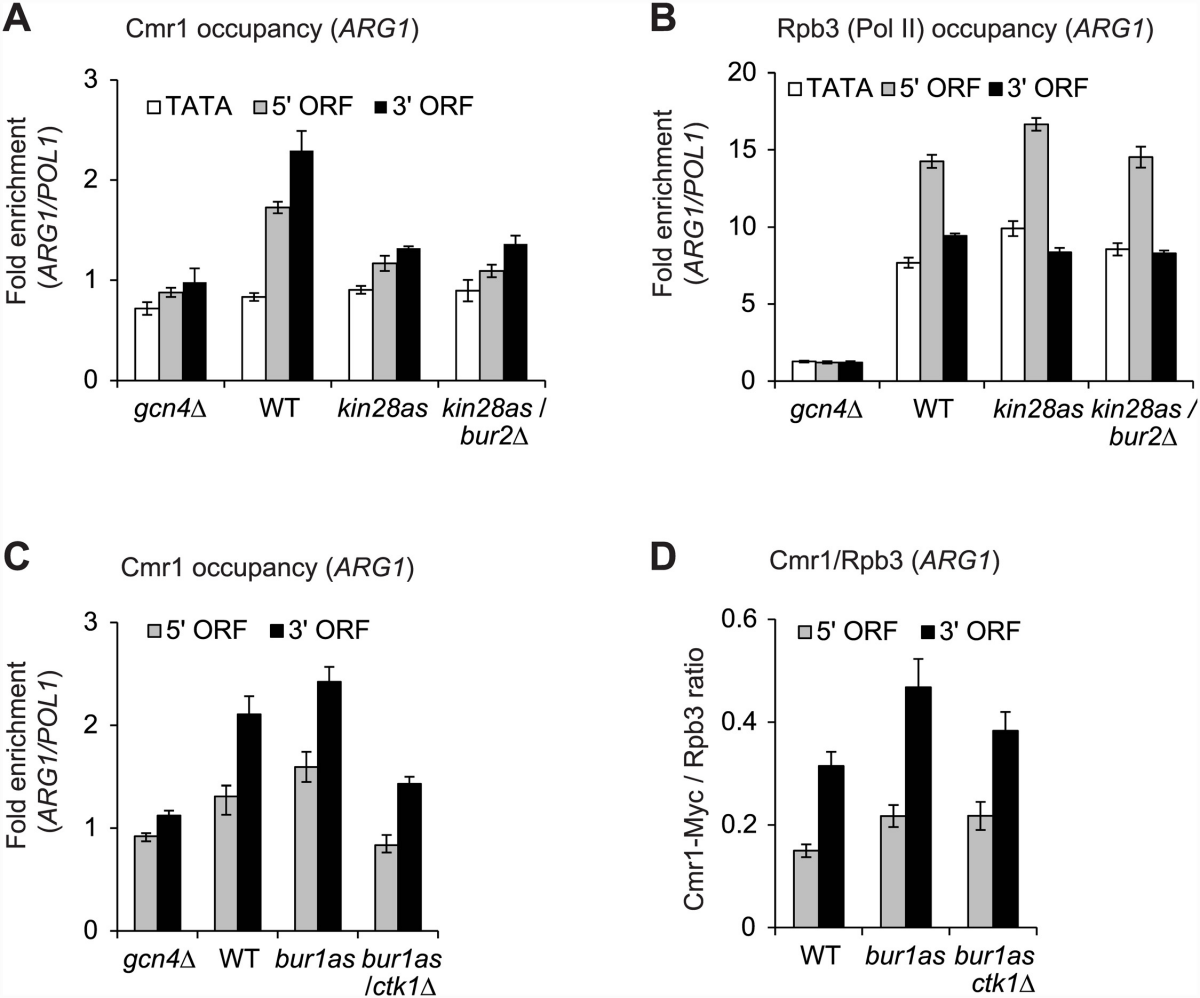
Pol II CTD kinase Kin28 stimulates Cmr1 recruitment to *ARG1* coding regions. A-C) Cmr1-Myc tagged strains (WT, *gcn4*Δ, *kin28as* and *kin28as*/*bur2*Δ) were treated with an ATP-analog NA-PP1 to inactivate Kin28 kinase activity, and Cmr1-Myc and Rpb3 (Pol II subunit) occupancies were determined by ChIP. Occupancies of Cmr1 (A), and Rpb3 (B) at the *ARG1* TATA, 5’ and 3’ ORFs are shown for the indicated strains. C-D) Cmr1-Myc tagged strains (WT, *gcn4*Δ, *bur1as* and *bur1as*/*ctk1*Δ) were treated with ATP-analog 3MB-PP1 to inactivate Bur1 kinase activity, and Cmr1 and Rpb3 occupancies were measured by ChIP. Occupancies of Cmr1 (C), and Cmr1/Rpb3 ratios (D) at the 5’ and 3’ ORFs of *ARG1* are shown for the indicated strains.

As expected, the kinase mutants showed substantially reduced Ser5P occupancy upon treatment with the ATP-analog NA-PP1 (S2A Fig) [17]. Interestingly, Cmr1 occupancy was also reduced, nearly to the levels observed in the *gcn4*Δ cells, at the *ARG1* 5’ and 3’ ORFs in both *kin28as* and *kin28as*/*bur2*Δ mutants (Fig 3A). However, WT level of Pol II occupancy was observed at *ARG1* in these mutants (Fig 3B), suggesting that the impaired Cmr1 occupancy, in the kinase mutants, is unlikely due to a Pol II binding defect at *ARG1*. Taken together, these results strongly suggest that Ser5 phosphorylation promotes Cmr1 recruitment at *ARG1*. Since no further reduction in Cmr1 occupancy was observed in the *kin28as*/*bur2*Δ mutant compared to the *kin28as* single mutant (Fig 3A), our results suggest that phosphorylation of Ser2 by Bur1/Bur2 does not stimulate Cmr1 recruitment.

To directly test the last conclusion, we examined Cmr1 occupancy in Ser2 kinase mutants, *bur1as* and *bur1as*/*ctk1*Δ. Bur1 along with Ctk1 phosphorylates Ser2 residue of the Pol II CTD (Ser2P) at the 5’ ends of transcribed genes, whereas Ctk1 has a major role in phosphorylating Ser2 at 3’ ends [37]. Strains containing a *bur1as* allele were treated with ATP-analog 3MB-PP1 to inactivate Bur1 kinase activity. Such treatment led to an expected reduction in Ser2P at the 5’ *ARG1* ORF in the *bur1as* mutant and a substantial reduction at both 5’ and 3’ ORFs in *bur1as*/*ctk1*Δ cells (*data not shown*, [23]). While, Cmr1 occupancy in the *bur1as* mutant was similar to the WT cells, it was reduced in *bur1as*/*ctk1*Δ (Fig 3C). However, Rpb3 occupancy in the *ARG1* ORF was also reduced in the double mutant (S2B Fig). As such, we calculated Cmr1/Rpb3 ratios (Fig 3D) and found that most of the reduction in Cmr1 occupancy at *ARG1* stems from the impaired Pol II binding in the Ser2 kinase double mutant. Similar conclusions were reached by examining the effect of *ctk1*Δ on Cmr1 recruitment (*data not shown*). Collectively, our results indicate that Ser5 phosphorylation by Kin28, but not of Ser2 by Bur1 and Ctk1, stimulates Cmr1 recruitment to the *ARG1* coding sequences.

### HDACs Rpd3 and Hos2 are important for Cmr1 association with coding regions

**Fig 4:**
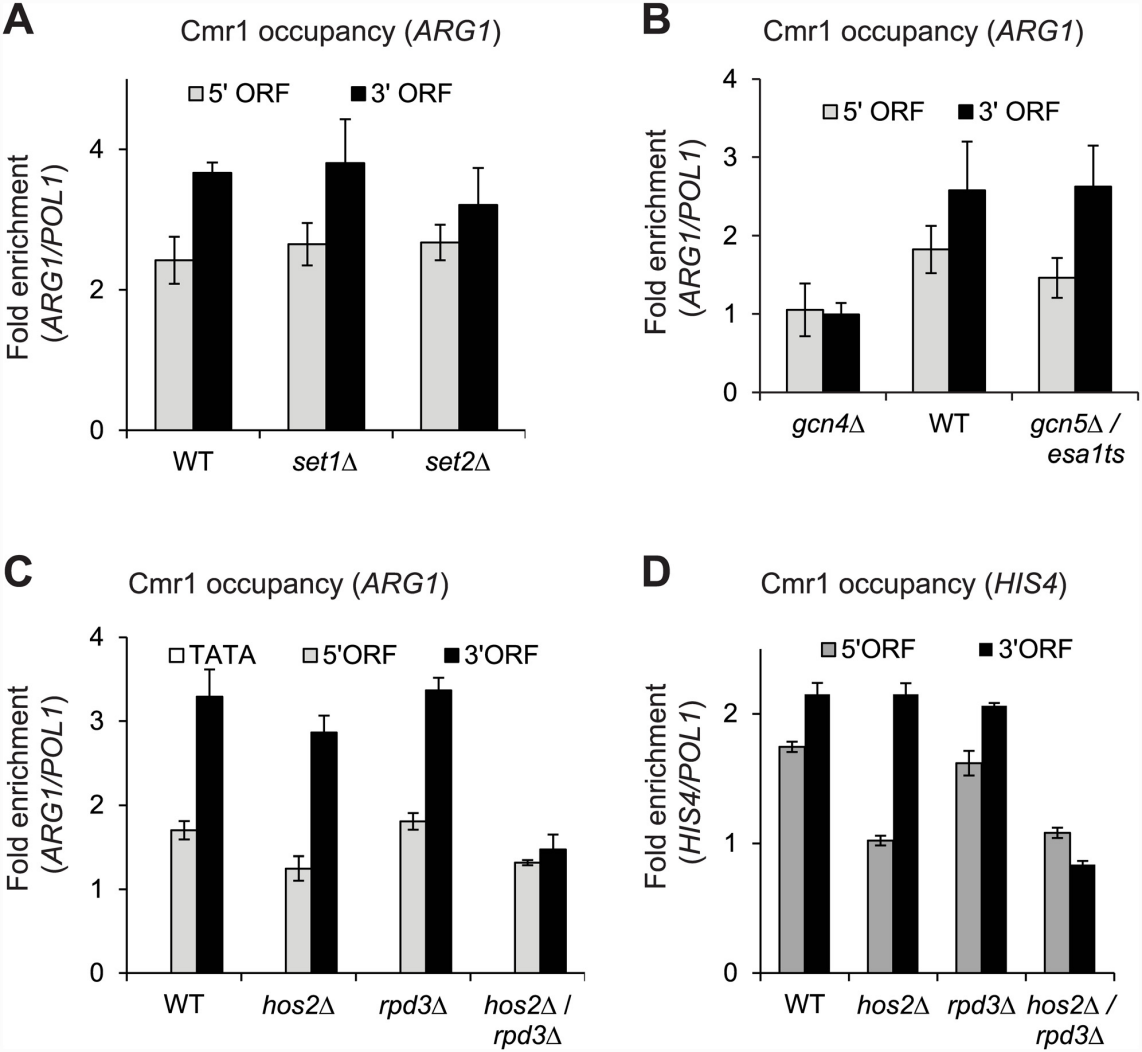
HDACs Rpd3 and Hos2 promote Cmr1 binding in the *ARG1* coding regions. A) Cmr1 occupancy in the WT and histone methyltransferase mutants (*set1*Δ and *set2*Δ) at *ARG1* 5’ and 3’ ORFs is shown. B) Occupancy of Cmr1 at the induced *ARG1* gene in WT, *gcn4*Δ and histone acetyltransferase mutant (*gcn5*Δ/*esa1ts*) is shown. C-D) Cmr1 ChIP occupancies in the induced WT and in histone deacetylase (HDACs) mutants (*hos2*Δ, *rpd3*Δ, and *hos2*Δ/*rpd3*Δ) at the ORFs of *ARG1* (C) and *HIS4* (D) are shown.

Having observed that the Ser5 kinase kin28 is required for recruiting Cmr1, we next investigated whether chromatin modifications play a role in regulating Cmr1 occupancy considering that Cmr1 interacts with histones [8]. To this end, we examined Cmr1 occupancy in mutants affecting histone modifications. The H3 N-terminal tails are methylated at lysine 4 (H3K4) and at lysine 36 (H3K36) by histone methyltransferases Set1 and Set2, respectively [39, 40]. Cmr1 occupancy in the *ARG1* coding region was unaffected in *set1*Δ and *set2*Δ mutants (Fig 4A), indicating that histone methylation is not important for recruitment or retention of Cmr1 over the coding regions of *ARG1*. We next examined the role of histone acetylation in modulating Cmr1 recruitment to the coding regions. Histone H3 and H4 N-terminal tails are acetylated by Gcn5-containing SAGA and Esa1-containing NuA4 histone acetyltransferase (HAT) complexes [41, 42]. Comparable occupancy of Cmr1 in the Gcn5 and Esa1 double mutant (*gcn5*Δ/*esa1ts*) and WT cells was observed in the *ARG1* ORF (Fig 4B) suggesting that histone acetylation is not critical in regulating Cmr1 occupancy.

To further test the role of histone acetylation, we analyzed Cmr1 occupancy in histone deacetylase (HDAC) mutants. Both Hos2-Set3 and Rpd3 HDACs are recruited via phosphorylated Pol II CTD [18, 43], recognize dimethylated (me2) H3K4 and H3K36, respectively and subsequently, deacetylate ORF nucleosomes [44–46]. No significant reduction in Cmr1 occupancy in the *ARG1* ORF was observed in *hos2*Δ or *rpd3*Δ strains (Fig 4C). Interestingly, a substantial reduction in Cmr1 occupancy was seen at the 3’ end of *ARG1* in the double mutant *rpd3*Δ/*hos2*Δ (Fig 4C), suggesting that HDACs Rpd3 and Hos2 redundantly promote Cmr1 recruitment to the 3’ end of *ARG1*. Similarly, we observed a greater reduction in Cmr1 occupancy at the *HIS4* ORF in the double mutant than in the respective single mutants (Fig 4D). These results thus indicate that Rpd3 and Hos2, in addition to Kin28, regulate association of Cmr1 to coding regions of Gcn4-regulated genes.

### Cmr1 does not regulate histone occupancy or transcription-coupled histone modifications

**Fig 5:**
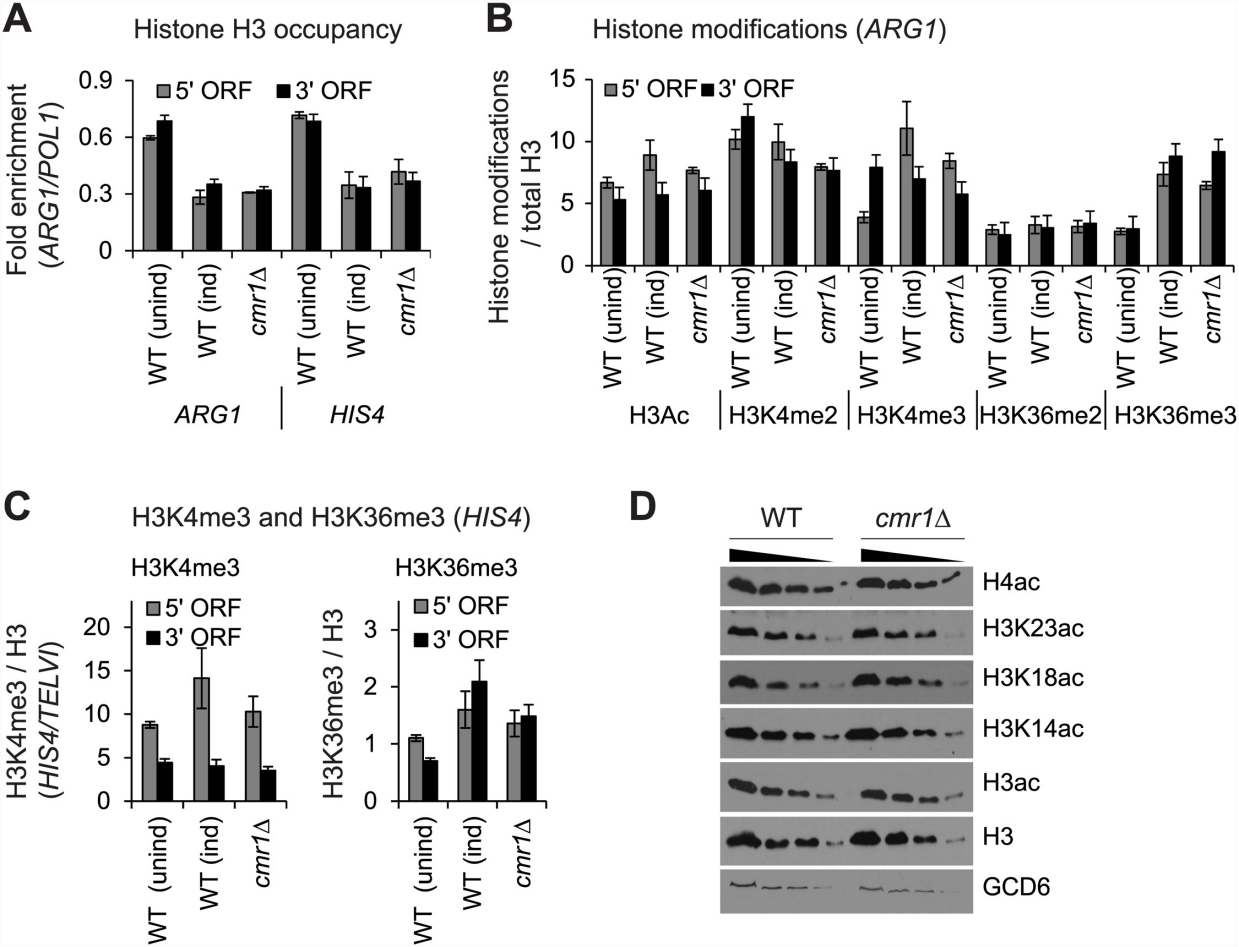
Cmr1 does not regulate histone occupancy or cotranscriptional histone modifications. WT and *cmr1*Δ strains were treated with SM to induce *ARG1* transcription and occupancies of histone H3 and histone modifications were determined by ChIP. A) H3 ChIP occupancies at *ARG1* and *HIS4* in SM-induced WT and *cmr1*Δ cells, and in the non-induced WT cells are shown. B) Occupancies of histone H3 modifications (acetylation, Ac; methylation, me; 2, di; 3, tri) normalized to H3 occupancy at *ARG1* in SM-induced WT and *cmr1*Δ strains are shown. The levels of these modifications for an un-induced WT strain are also shown. C) Trimethylated (me3) H3K4 and K3K36 occupancies normalized to H3 levels at *HIS4* ORFs in the WT and *cmr1*Δ cells. D) Whole cell extracts were prepared from WT and *cmr1*Δ cells and H3 acetylation levels were detected by western blot. H3 and Gcd6 (a translational factor) were used as loading controls.

Having observed recruitment of Cmr1 to the transcribed regions (Figs 1 and 2) and considering that it has been identified as a histone binding protein [8], we asked whether Cmr1 might regulate histone occupancy. As such, we examined histone H3 occupancy at *ARG1* and *HIS4* in WT and *cmr1*Δ cells. While H3 occupancies at the 5’ and 3’ ORFs of *ARG1* and *HIS4* decreased upon induction (Fig 5A), as expected [18], both induced WT and *cmr1*Δ cells exhibited very similar H3 occupancies in the *ARG1* ORFs. These results, therefore, suggest that Cmr1 is dispensable for histone eviction and for maintaining normal histone occupancy levels in transcribed coding sequences.

Next, we examined ORF-associated histone modifications in the induced WT and *cmr1*Δ cells. A non-induced WT strain was included in the analysis to ascertain that expected changes in histone modifications occur upon induction. Only a small increase in H3 acetylation (H3Ac) was observed in the *ARG1* ORF upon induction in WT cells (Fig 5B), consistent with a previous study showing that *ARG1* ORF nucleosomes are rapidly deacetylated by multiple HDACs that are recruited to the *ARG1* ORF [18]. However, under inducing conditions, *cmr1*Δ did not elicit any significant change in H3Ac levels at the *ARG1* ORF compared to the induced WT cells (Fig 5B). In contrast to H3Ac, induction led to ~ 3 fold increase in the level of tri-methylated (me3) H3K4 and H3K36 at the 5’ and 3’ regions of *ARG1* ORF, respectively. These last results are consistent with published studies showing that transcriptionally active genes have the greatest levels of H3K4me3 at the 5’ ends and H3K36me3 at the 3’ ends [47]. Unlike differential levels of trimethylation at the 5’ and 3’ ends, uniform dimethylated H3K4 and H3K36 (me2) levels were observed in the *ARG1* ORF (Fig 5B). However, methylated H3K36 and H3K4 occupancies were similar at the *ARG1* ORF in the induced WT and *cmr1*Δ mutant. These results suggest that Cmr1 does not regulate cotranscriptional histone acetylation or methylation. Similar conclusions were reached upon examining H3K4me3 and H3K36me3 in the coding regions of *HIS4*, which recruits Cmr1 upon induction (Fig 5C).

These conclusions were further supported by analyzing the level of histone modifications, by immunoblotting, in whole-cell extracts prepared from the WT and *cmr1*Δ cells. As shown in Fig 5D, both acetylated H3 and acetylated H4 levels were similar in the WT and *cmr1*Δ mutant. Similarly, no significant changes were observed when individual acetylated H3 residues (K14, K18 and K23) (Fig 5D) or methylated H3K4 and H3K36 (*data not shown*) levels were analyzed in WT and *cmr1*Δ. Overall, these results indicate that while Cmr1 interacts with histones, it may not regulate their occupancy or modifications that are generally associated with transcription activation.

### Cmr1 regulates Pol II occupancy in transcribed coding regions genome-wide

**Fig 6:**
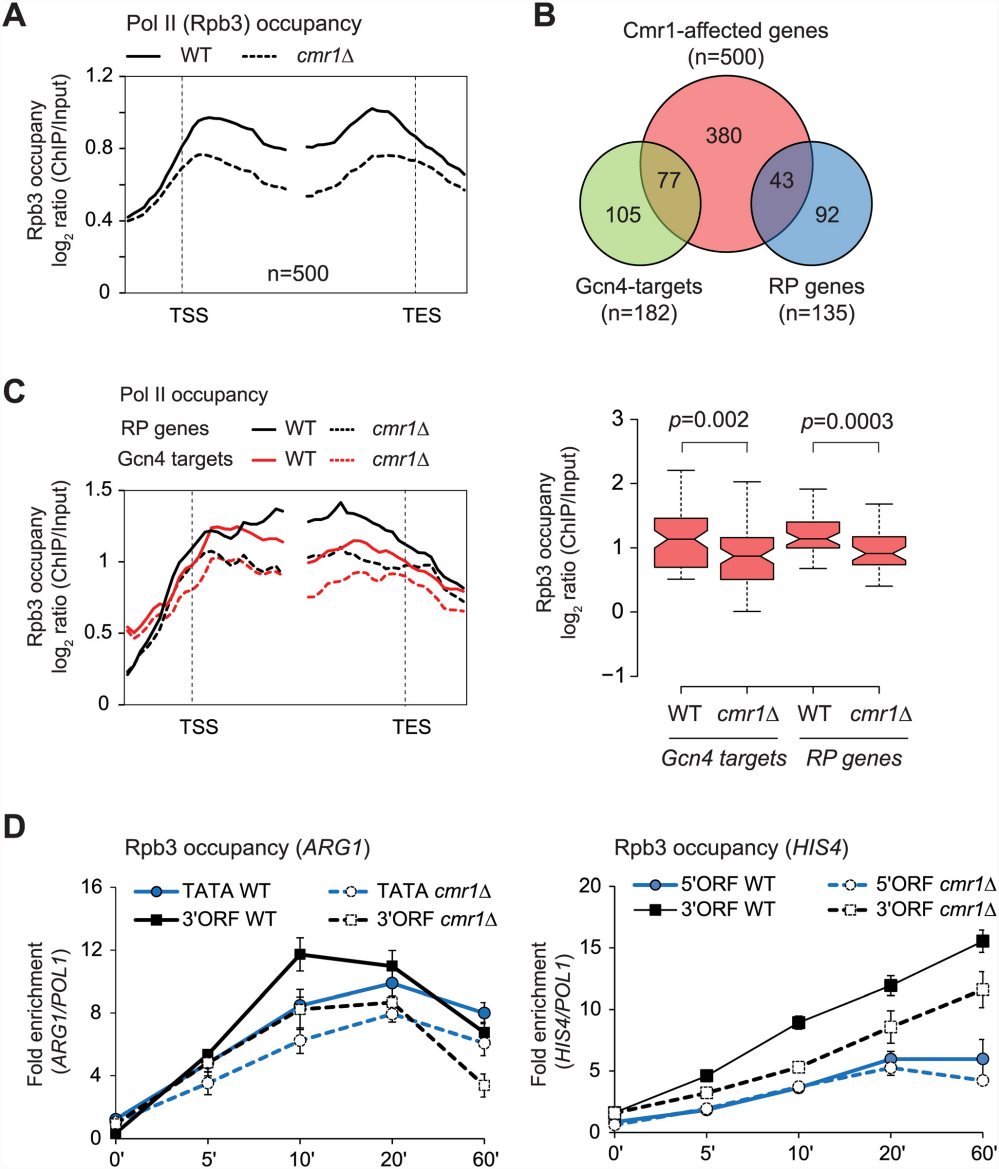
Cmr1 promotes transcription of *ARG1* and *HIS4*. A) Pol II occupancy profile for the 500 genes (among those genes harboring average Rpb3 occupancies >0.5 log_2_ ratio) showing the greatest Rpb3 binding defect in *cmr1*Δ cells. B) Venn diagram showing enrichment of Gcn4 and RP (ribosomal protein) genes among the top 500 Cmr1-affected genes. C) Rpb3 enrichments at the Gcn4 (n = 77) and RP genes (n = 43) among the top 500 Cmr1-affected genes (left) and gene average profile (right) in WT and *cmr1*Δ are shown. Notches represent 95% confidence intervals for each median. D) Rpb3 occupancy at the indicated regions of *ARG1* (left) and *HIS4* (right) was determined at various time-points during induction by ChIP in the WT and *cmr1*Δ strain.

We next asked whether Cmr1 is important for transcription. To address this, we determined Rpb3 (Pol II subunit) occupancy in a *cmr1*Δ mutant under amino acid starvation conditions, genome-wide. Average gene Rpb3 occupancy profile for the 500 most-affected genes (Cmr1-affected), among transcribing genes (Rpb3 log_2_ ratio > 0.5), is shown in Fig 6A. It is interesting that *cmr1*Δ elicits greatest Pol II reduction downstream of the TSS at these genes, suggesting that Cmr1 may regulate transcription at the elongation step. This group of genes included 77 gcn4-regulated (hypergeometric p-value = 9.6 x 10^−35^) and 50 ribosomal protein genes (RP genes; hypergeometric p-value = 2.4 x 10^−14^) (Fig 6B). Pol II occupancy defect at these genes in the *cmr1*Δ mutant was also localized primarily in the coding sequences (Fig 6C). These results are consistent with our earlier results (Fig 2) showing that Cmr1 is recruited to the coding regions of many Gcn4 targets and RP genes.

To confirm our ChIP-chip results, we examined Rpb3 ChIP occupancy at *ARG1* and *HIS4* at different time points during their induction by SM. As expected, in the WT cells, Pol II occupancy gradually increased both at the TATA and 3’ ORF of *ARG1* upon SM treatment and exhibited the maximum recruitment between the 10 and 20 minutes time points (Fig 6D, left), in agreement with a previous study [26]. While *cmr1*Δ evoked a smaller reduction in Pol II occupancy at *ARG1* TATA, even at an early time point (5 minutes post-induction), a greater reduction in Pol II binding was observed at the 3’ end of *ARG1*, suggesting that Cmr1 plays a role in the elongation phase of transcription. Since *ARG1* is a relatively short gene (1263 base pairs), we examined Rpb3 occupancy at *HIS4*, which has a longer coding region (2400 base pairs). At the *HIS4* 5’ ORF, Pol II occupancy in *cmr1*Δ was similar to that observed in the WT cells at all of the time-points examined (Fig 6D, right) suggesting that Cmr1 is likely dispensable for initial recruitment of Pol II or promoter clearance at this gene. However, similar to that observed at *ARG1*, Pol II occupancy was reduced at the *HIS4* 3’ ORF in *cmr1*Δ, supporting a role for Cmr1 in the elongation step of transcription (Fig 6D, right).

Collectively, our results indicate that Cmr1 recruitment to the coding regions stimulates transcription of many highly expressed genes. Although it was identified as a key histone-interacting protein, it is largely dispensable for regulating histone occupancy or cotranscriptional histone modifications.

## Discussion

In this study, we have examined a role for Cmr1 in regulating transcription of Pol II transcribed genes. We show that Cmr1 is recruited to coding sequences in a transcription dependent manner to many genes, genome-wide, including ribosomal protein genes and those induced by gcn4 and Gal4 transcriptional activators. The recruitment of this protein is stimulated by the Pol II CTD kinase Kin28 as well as by Rpd3 and Hos2 containing histone deacetylase complexes. We also show that Cmr1 is important for maintaining WT occupancies of Pol II at the coding sequences of many transcribed genes.

### Cmr1 localizes to the coding regions

Cmr1 is a highly conserved protein that was recently identified to be strongly associated with all four histones as well as with several transcription factors, including histone deacetylases, RSC chromatin remodeler and Paf1 complex, which are generally linked to Pol II transcribed genes [8]. Interestingly, no interactions with Pol II subunits were detected suggesting that Cmr1 may regulate transcription of Pol II genes. While deletion of Cmr1 altered expression of ~400 genes, including many Pol II genes, genome-wide, it was not clear whether Cmr1 regulates expression by acting at transcription initiation or at post-initiation steps of transcription [8]. Our ChIP analysis of Cmr1 occupancy revealed that it preferentially associates with transcribed coding regions of Gcn4 and Gal4 targets, but not with promoter regions (Fig 1A and 1G). Furthermore, Cmr1 occupancy in coding regions was highest at the time-point corresponding to maximal recruitment of Pol II (Figs 1B and 5A), indicating that Cmr1 is actively recruited to ORFs in a transcription-dependent manner. Consistent with this idea, many strongly transcribed genes displayed Cmr1 enrichment in their coding sequences, genome-wide. These Cmr1-enriched genes included Gcn4 targets (strongly expressed under amino acid starvation) as well as ribosomal protein genes, which are among the most highly expressed genes (Fig 2). However, a few highly transcribed genes, including *ADH1* and *PMA1*, failed to show Cmr1 occupancy in their coding sequences (Fig 2A). It is possible that Cmr1 may only be transiently associated with these genes, and thereby escapes detection by ChIP. Chromatin remodeling complex RSC displayed a similar trend, in that it localizes to many, but not all, highly transcribing genes [19]. Interestingly, RSC was identified as a Cmr1-interacting remodeler [8]. Enrichment of Cmr1 specifically in ORFs of many transcribed genes, strongly suggests a role for Cmr1 in regulating post-initiation steps of transcription.

### How is Cmr1 localized to the coding sequences?

Our results suggest a role for Ser5 Pol II CTD kinase Kin28 in stimulating Cmr1 recruitment to the coding regions (Fig 3). The phosphorylation of Ser5 marks the transition from initiating to elongating polymerase. Ser5 phosphorylation has been shown to stimulate recruitment of many factors that regulate transcription elongation-coupled events [32]. For example, Ser5 phosphorylation promotes recruitment of capping enzymes [48–50], HAT complexes SAGA and NuA4 [17, 51], histone deacetylase complexes Rpd3S and Hos2-Set3 [18, 43], Paf1 complex [38] and others [32, 52, 53]. Our finding that Ser5 phosphorylation stimulates Cmr1 recruitment to *ARG1* ORF is consistent with the role of phosphorylated Pol II CTD in coordinating elongation factor recruitment. However, Cmr1 occupancy was not affected in the *ARG1* 3’ ORF in Ser2 kinase mutants, suggesting that Cmr1 is brought to coding sequences during early elongation steps. Considering that Cmr1 is a histone-associated protein [8], histone modifications could also promote Cmr1 association with chromatin. Histone acetylation, particularly in coding regions, is highly dynamic, and multiple HATs and HDACs are recruited to maintain appropriate levels of histone acetylation in ORF sequences [17, 18, 54]. While acetylation of ORF nucleosomes promotes transcription elongation [17, 19], these acetylation marks are erased by HDACs to prevent aberrant transcription [46, 55, 56]. The role for HDACs (Rpd3 and Hos2) in promoting Cmr1 recruitment is reminiscent of a role for these HDACs in stimulating Spt6 occupancy in coding regions [23]. Furthermore, the requirement of Hos2 and Rpd3 for Cmr1 recruitment is in agreement with previous reports showing that Cmr1, Rpd3 and Hos2 co-localize to the same sub-nuclear focus upon genotoxic stress [5, 6]. Collectively, our results indicate that Ser5 phosphorylation by Kin28 and histone deacetylases but not Ser2 phosphorylation, histone methylation or histone acetylation by HATs, are needed for maintaining high-level occupancy of Cmr1 in coding regions.

### Why does Cmr1 localize to coding regions?

Cmr1 enrichment primarily at transcribed coding sequences, including those of Gcn4 and Gal4 target genes, suggests that Cmr1 might function during the elongation phase of transcription. Although, Cmr1 was identified as one of the core histone binding proteins, deleting *CMR1* did not alter the pattern or the levels of histone acetylation or methylation—the two histone modifications that are generally associated with transcribing genes (Fig 5). However, in the *cmr1*Δ cells, several genes exhibited lower Pol II occupancies in their coding sequences, particularly downstream of the TSS, implying that Cmr1 may promote Pol II traversal through coding regions (Fig 6A–6C). Diminished Pol II ORF occupancies were also observed during the course of induction of *ARG1* and *HIS4* in the *cmr1*Δ mutant. It is interesting to note that strongest reduction in Pol II occupancies was observed at the 3’ ORFs of both genes (Fig 6D). These results suggest that Cmr1 acts at the elongation step and are consistent with of the higher Cmr1 occupancy in the 3’ ends of these genes. However, the detailed mechanism by which Cmr1 promotes transcription remains to be determined. Proteomic analyses have indicated that Cmr1 interacts with chromatin remodeling complexes RSC and SWI/SNF, and histone chaperone FACT (Spt16/Pob3) subunits [8]. FACT, as well as chromatin remodelers RSC and SWI/SNF localize to coding regions and promote transcription [19, 57, 58]. It is plausible that Cmr1 may regulate recruitment or function of these chromatin remodelers or histone chaperones, and aid in efficient elongation of Pol II. Collectively, our study provides evidence for a role of Cmr1 in regulating transcription by directly associating with the transcribed coding regions.

## Supporting Information

S1 Fig Rpb3 occupancy at *GAL1*. *GAL1* transcription was induced by treating cells grown in raffinose (Raf) with 2% galactose (Gal) and repressed by adding 4% glucose (Glu). Rpb3 occupancy at the *GAL1* TATA, 5’ and 3’ ORFs is shown.
(EPS)

S2 Fig Ser5P and Pol II occupancy at *ARG1*. A) The WT, *gcn4*Δ, *kin28as* and *kin28as*/*bur2*Δ cells were treated with an ATP-analog NA-PP1 to inactivate Kin28 activity, and the occupancy of the Pol II CTD phosphorylated at Ser5 (Ser5P) was determined at the *ARG1* TATA, 5’ ORF and 3’ ORF by ChIP. B) The WT, *gcn4*Δ, *bur1as* and *bur1as*/*ctk1*Δ cells were treated with an ATP-analog 3MB-PP1 to inactivate Bur1, and Rpb3 occupancy at the *ARG1* 5’ and 3’ ORFs was determined by ChIP.
(EPS)

S1 Table Yeast Strains used in this study. (DOCX)

S2 Table Primers used for ChIP experiments. (DOCX)
