# Supplementary material for: Recruitment of Saccharomyces cerevisiae Cmr1/Ydl156w to Coding Regions Promotes Transcription Genome Wide
Source: PLoS One. 2016 Feb 5;11(2):e0148897. doi: 10.1371/journal.pone.0148897 (PMC4744024; doi:10.1371/journal.pone.0148897)
Supplement: S1 Table — (DOCX) [file pone.0148897.s003.docx]

**S1 Table: Yeast Strains used in this study:**

| **Strain** | **Parent** | **Genotype** | **Source or Reference** |
| --- | --- | --- | --- |
| ***Untagged*** | | | |
| yFR762 | S288C | *MAT*α *ade2*::*hisG his3*∆*200 leu2*∆*0 lys2*∆*0 met15*∆*0 trp1*∆*63 ura3*∆*0* | (Bataille *et al.* 2012) |
| yFR763 | S288C | *MATα ade2::hisG his3∆200 leu2∆0 lys2∆0 met15∆0 trp1∆63 ura3∆0kin28*::*kin28-L83G*  [pSH579 *ARS CEN URA3 kin28-L83c*] | (Bataille *et al.* 2012) |
| yFR912 | S288C | *MAT*α *ade2*::*hisG his3*∆*200 leu2*∆*0 lys2*∆*0 met15*∆*0 trp1*∆*63 ura3*∆*0kin28*::*kin28-L83G*∆*bur2*::*Leu2* [pSH579 *ARS CEN URA3 kin28-L83G* | (Bataille *et al.* 2012) |
| Y729 | BY4741 | *MAT*a *his3*∆*1 leu2*∆*0 met15*∆*0 ura3*∆*0* | (Qiu *et al.* 2009) |
| HQY1236 | BY4741 | *MAT*a *his3*∆*1 leu2*∆*0 met15*∆*0 ura3*∆*0 bur1as ctk1*∆::*kanMX4* | (Qiu *et al.* 2009) |
| Y64 (WT HDACs) | BY4705 | *MAT*α *ade2*∆::*hisG HIS3*∆*200 LEU2*∆*0 ura3*∆*0 trp1*∆*63 LYS2*∆*0 met15*∆*0* | (Sharma *et al.* 2007) |
| *hos2∆/rpd3∆* | BY4705 | *MAT*α *ade2*∆::*hisG HIS3*∆*200 LEU2*∆*0 ura3*∆*0 trp1*∆*63 LYS2*∆*0 met15*∆*0 rpd3*::*LEU2*  *hos2*::*LYS2 CMR1-13myc*::*HIS3* | (Sharma *et al.* 2007) |
| *hos2∆* | BY4705 | *MAT*α *ade2*∆::*hisG HIS3*∆*200 LEU2*∆*0 ura3*∆*0 trp1*∆*63 LYS2*∆*0 met15*∆*0 hos2*::*LYS2* | (Govind *et al.* 2010) |
| *rpd3∆* | BY4705 | *MAT*α *ade2*∆::*hisG HIS3*∆*200 LEU2*∆*0 ura3*∆*0 trp1*∆*63 LYS2*∆*0 met15*∆*0 rpd3*::*LEU2 CMR1-13myc*::*HIS3* | (Govind *et al.* 2010) |
| *cmr1∆* | BY4741 | *MAT*a *his3*∆*1 leu2*∆*0 met15*∆*0 ura3*∆*0* *cmr1∆* :: *kanMX4* | Thermo  Scientific |
| *gcn4∆* | BY4741 | *MAT*a *his3*∆*1 leu2*∆*0 met15*∆*0 ura3*∆*0* *gcn4Δ :: kanMX4* | Thermo  Scientific |
| *Paf1∆* | BY4741 | *MAT*a *his3*∆*1 leu2*∆*0 met15*∆*0 ura3*∆*0* *paf1Δ :: kanMX4* | Thermo  Scientific |
| *gcn5∆/esa1ts* | DG154 | *MATa his3Δ leu2Δ met15Δ ura3Δ gcn5Δ::kanMX4 esa1L254P* | (Ginsburg *et al.* 2009) |
| *set1∆* | BY4741 | *MAT*a *his3*∆*1 leu2*∆*0 met15*∆*0 ura3*∆*0* *set1∆* :: *kanMX4* | Thermo  Scientific |
| *set2∆* | BY4741 | *MAT*a *his3*∆*1 leu2*∆*0 met15*∆*0 ura3*∆*0* *set2∆* :: *kanMX4* | Thermo  Scientific |
| ***Myc Tagged*** | | | |
| JJY1 | yFR762 | *MAT*α *ade2*::*hisG his3*∆*200 leu2*∆*0 lys2*∆*0 met15*∆*0 trp1*∆*63 ura3*∆*0 CMR1-13myc*::*HIS3* | This Study |
| JJY2 | yFR763 | *MATα ade2::hisG his3∆200 leu2∆0 lys2∆0 met15∆0 trp1∆63 ura3∆0kin28*::*kin28-L83G*  [pSH579 *ARS CEN URA3 kin28-L83c*] *CMR1 -13myc*::*HIS3* | This Study |
| JJY3 | yFR912 | *MAT*α *ade2*::*hisG his3*∆*200 leu2*∆*0 lys2*∆*0 met15*∆*0 trp1*∆*63 ura3*∆*0kin28*::*kin28-L83G*∆*bur2*::*Leu2* [pSH579 *ARS CEN URA3 kin28-L83G*] *CMR1-13myc*::*HIS3* | This Study |
| JJY4 | BY4741 | *MAT*a *his3*∆*1 leu2*∆*0 met15*∆*0 ura3*∆*0 CMR1-13myc*::*HIS3* | This Study |
| JJY5 | BY4741 | *MAT*a *his3*∆*1 leu2*∆*0 met15*∆*0 ura3*∆*0 bur1as ctk1*∆::*kanMX4 CMR1-13myc*::*HIS3* | This Study |
| JJY6 | BY4705 | *MAT*α *ade2*∆::*hisG HIS3*∆*200 LEU2*∆*0 ura3*∆*0 trp1*∆*63 LYS2*∆*0 met15*∆*0CMR1-13myc*::*HIS3* | This Study |
| JJY7 | BY4705 | *MAT*α *ade2*∆::*hisG HIS3*∆*200 LEU2*∆*0 ura3*∆*0 trp1*∆*63 LYS2*∆*0 met15*∆*0 rpd3*::*LEU2 hos2*:: *LYS2 CMR1-13myc*::*HIS3* | This Study |
| PS1 | BY4705 | *MAT*α *ade2*∆::*hisG HIS3*∆*200 LEU2*∆*0 ura3*∆*0 trp1*∆*63 LYS2*∆*0 met15*∆*0 hos2*::*LYS2* | This Study |
| JJY8 | BY4705 | *MAT*α *ade2*∆::*hisG HIS3*∆*200 LEU2*∆*0 ura3*∆*0 trp1*∆*63 LYS2*∆*0 met15*∆*0 rpd3*::*LEU2 CMR1-13myc*::*HIS3* | This Study |
| JJY9 | BY4741 | *MAT*a *his3*∆*1 leu2*∆*0 met15*∆*0 ura3*∆*0* *gcn4Δ :: kanMX4 CMR1-13myc*::*HIS3* | This Study |
| JJY10 | BY4741 | *MAT*a *his3*∆*1 leu2*∆*0 met15*∆*0 ura3*∆*0 HOS2-13myc*::*HIS3* | This Study |
| JJY11 | BY4741 | *MAT*a *his3*∆*1 leu2*∆*0 met15*∆*0 ura3*∆*0* *cmr1∆* ::*kanMX4 HOS2-13myc*::*HIS3* | This Study |
| JJY12 | BY4741 | *MAT*a *his3*∆*1 leu2*∆*0 met15*∆*0 ura3*∆*0 RCO1-13myc*::*HIS3* | This Study |
| JJY13 | BY4741 | *MAT*a *his3*∆*1 leu2*∆*0 met15*∆*0 ura3*∆*0* *cmr1∆* ::*kanMX4 RCO1-13myc*::*HIS3* | This Study |
| JJY14 | DG154 | *MATa his3Δ leu2Δ met15Δ ura3Δ gcn5Δ::kanMX4 esa1L254P CMR1-myc13::HIS3* | This Study |
| JJY15 | BY4741 | *MAT*a *his3*∆*1 leu2*∆*0 met15*∆*0 ura3*∆*0* *paf1Δ :: kanMX4 CMR1-myc13::HIS3* | This Study |
| JJY16 | BY4741 | *MAT*a *his3*∆*1 leu2*∆*0 met15*∆*0 ura3*∆*0 SPT16-13myc*::*HIS3* | This Study |
| JJY17 | BY4741 | *MAT*a *his3*∆*1 leu2*∆*0 met15*∆*0 ura3*∆*0* *cmr1∆* ::*kanMX4 SPT16-13myc*::*HIS3* | This Study |
| JJY18 | BY4741 | *MAT*a *his3*∆*1 leu2*∆*0 met15*∆*0 ura3*∆*0 SPT6-13myc*::*HIS3* | This Study |
| JJY19 | BY4741 | *MAT*a *his3*∆*1 leu2*∆*0 met15*∆*0 ura3*∆*0* *cmr1∆* ::*kanMX4 SPT6-13myc*::*HIS3* | This Study |
| JJY20 | BY4741 | *MAT*a *his3*∆*1 leu2*∆*0 met15*∆*0 ura3*∆*0* *set1∆* :: *kanMX4 CMR1-myc13::HIS3* | This Study |
| JJY21 | BY4741 | *MAT*a *his3*∆*1 leu2*∆*0 met15*∆*0 ura3*∆*0* *set2∆* :: *kanMX4 CMR1-myc13::HIS3* | This Study |
| PSY2 | BY4741 | *MAT*a *his3*∆*1 leu2*∆*0 met15*∆*0 ura3*∆*0 RTF1-13myc*::*HIS3* | This Study |
| PSY3 | BY4741 | *MAT*a *his3*∆*1 leu2*∆*0 met15*∆*0 ura3*∆*0* *cmr1∆* ::*kanMX4 RTF1-13myc*::*HIS3* | This Study |
| JJY22 | BY4741 | *MAT*a *his3*∆*1 leu2*∆*0 met15*∆*0 ura3*∆*0 SPT4-13myc*::*HIS3* | This Study |
| JJY23 | BY4741 | *MAT*a *his3*∆*1 leu2*∆*0 met15*∆*0 ura3*∆*0* *cmr1∆* ::*kanMX4 SPT4-13myc*::*HIS3* | This Study |

**References**

Bataille, A. R., C. Jeronimo, P. E. Jacques, L. Laramee, M. E. Fortin *et al.*, 2012 A universal RNA polymerase II CTD cycle is orchestrated by complex interplays between kinase, phosphatase, and isomerase enzymes along genes. Mol Cell 45**:** 158-170.

Ginsburg, D. S., C. K. Govind and A. G. Hinnebusch, 2009 NuA4 Lysine Acetyltransferase Esa1 Is Targeted to Coding Regions and Stimulates Transcription Elongation with Gcn5. Mol. Cell. Biol. 29**:** 6473-6487.

Govind, C. K., H. Qiu, D. S. Ginsburg, C. Ruan, K. Hofmeyer *et al.*, 2010 Phosphorylated Pol II CTD recruits multiple HDACs, including Rpd3C(S), for methylation-dependent deacetylation of ORF nucleosomes. Mol Cell 39**:** 234-246.

Qiu, H., C. Hu and A. G. Hinnebusch, 2009 Phosphorylation of the Pol II CTD by KIN28 Enhances BUR1/BUR2 Recruitment and Ser2 CTD Phosphorylation Near Promoters. Molecular Cell 33**:** 752-762.

Sharma, V. M., R. S. Tomar, A. E. Dempsey and J. C. Reese, 2007 Histone deacetylases RPD3 and HOS2 regulate the transcriptional activation of DNA damage-inducible genes. Molecular and Cellular Biology 27**:** 3199-3210.
