# Supplementary material for: Recruitment of Saccharomyces cerevisiae Cmr1/Ydl156w to Coding Regions Promotes Transcription Genome Wide
Source: PLoS One. 2016 Feb 5;11(2):e0148897. doi: 10.1371/journal.pone.0148897 (PMC4744024; doi:10.1371/journal.pone.0148897)
Supplement: S2 Table — (DOCX) [file pone.0148897.s004.docx]

**S2 Table. Primers used for ChIP experiments**

| **Gene** | **Region** | **Orientation** | **Primer Sequence (5' - 3')** |
| --- | --- | --- | --- |
| *ARG1* | TATA | Forward | TAATCTGAGCAGTTGCGAGA |
| *ARG1* | TATA | Reverse | ATGTTCCTTATCGCTGCACA |
| *ARG1* | 5ORF | Forward | TGGCTTATTCTGGTGGTTTAG |
| *ARG1* | 5ORF | Reverse | ATCCACACAAACGAACTTGCA |
| *ARG1* | 3ORF | Forward | TTCTGGGCAGATCTACAAAGA |
| *ARG1* | 3ORF | Reverse | AAGTCAACTCTTCACCTTTGG |
| *His4* | 5ORF | Forward | AAGTTCCATTGGTGGCTTTG |
| *His4* | 5ORF | Reverse | TGATTGGAGAAAACACCGTTC |
| *His4* | 3ORF | Forward | GTCCGTATTTGTGGGTGCTT |
| *His4* | 3ORF | Reverse | GGGTAATGTTTTGGGCAGTG |
| *PMA1* | 5'ORF | Forward | CGACGACGAAGACAGTGATA |
| *PMA1* | 5'ORF | Reverse | ATTCTTTTTCGTCAGCCATTT |
| *PMA1* | 3 ORF | Forward | CGTGTCTGGATCTGGTCTAT |
| *PMA1* | 3 ORF | Reverse | GAAGTCTTCGACACTTCTGG |
| *POL1* | ORF | Forward | GACAAAATGAAGAAAATGCTGATGCACC |
| *POL1* | ORF | Reverse | TAATAACCTTGGTAAAACACCCTG |
| *TEL VI* | ORF | Forward | GCTGAGTTTAACGGTGATTATT |
| *TEL VI* | ORF | Reverse | CCAGTCCTCATTTCCATCAAT |
